# Promoting lifestyle change through text messages to patients with hypertension: A randomized controlled trial in Swedish primary care

**DOI:** 10.1016/j.pmedr.2025.103009

**Published:** 2025-02-17

**Authors:** Hanna Glock, Amanda Björk Javanshiri, Beata Borgström Bolmsjö, Ulf Jakobsson, Veronica Milos Nymberg, Moa Wolff, Susanna Calling

**Affiliations:** a Center for Primary Health Care Research, Department of Clinical Sciences Malmö, Lund University, Box 50332, 202 13 Malmö, Sweden; b Office for Primary Care, Skåne University Hospital, Lund, Sweden

**Keywords:** Text messaging, Hypertension, Health behavior, Lifestyle, Primary care, Randomized controlled trial, Theory of Planned Behavior

## Abstract

**Objective:**

We aimed to investigate whether health-promoting text messages sent to patients with hypertension in primary care could affect lifestyle habits, and if the Theory of Planned Behavior could be used to identify moderators of intervention effects.

**Methods:**

From September 2020 to December 2022, patients with hypertension were randomly selected from the patient register at 10 Swedish primary health care centers and randomized 1:1 to receive health-promoting text messages or treatment as usual (*N* = 401). The intervention group received four text messages per week for six months. Self-reported measures of lifestyle habits were collected through a questionnaire at baseline and after six months. Predictors of behavioral change according to the Theory of Planned Behavior were collected through a baseline questionnaire. The data were analyzed according to the intention-to-treat principle. We compared lifestyle habits between the intervention and control groups at follow-up with adjustment for baseline measures through logistic regression analysis and analysis of covariance.

**Results:**

The text message group had a statistically significant decrease in the proportion of participants with alcohol use above four standard drinks per week (OR 0.35, 95 % CI 0.15–0.81), and in the proportion of participants being physically inactive (OR 0.60, 95 % CI 0.37–0.98). The effect could not be explained or predicted by a pragmatic adaptation of the Theory of Planned Behavior.

**Conclusions:**

Health-promoting text messages could be offered to Swedish primary care patients with hypertension as part of the effort to improve their lifestyle habits.

## Highlights

• This trial assessed the effects of six months of health-promoting text messages.
• We analyzed lifestyle changes in primary care patients with hypertension.
• The text message recipients decreased alcohol use and increased physical activity.
• Ratings on motivational questions did not predict effects on lifestyle habits.

## 1 Introduction

High systolic blood pressure is the world's leading modifiable risk factor for cardiovascular disease and premature death (Vaduganathan et al., 2022). While medications are often needed, blood pressure and its cardiovascular effects can be substantially influenced by lifestyle habits (Barbaresko et al., 2018; Vaduganathan et al., 2022; Zhou et al., 2021). Non-pharmacological interventions have been shown to affect lifestyle habits and lower blood pressure, with a dietary approach indicated to be most effective, followed by exercise and comprehensive lifestyle modification (Bulto et al., 2024; Fu et al., 2020; Silva et al., 2024; Xun et al., 2023). Guidelines on hypertension have expressed a consensus regarding the importance of lifestyle habits (Maniero et al., 2023). Despite this, unhealthy lifestyle habits are common in European primary care patients with hypertension (Kotseva et al., 2021). It has been hypothesized that the number of patients at risk, in combination with limited health care resources, makes individually oriented lifestyle interventions provided by health care professionals a questionable effort (Albarqouni et al., 2023; Albarqouni et al., 2022). Consequently, more efficient lifestyle interventions that are readily applicable in primary care are needed.

Digital interventions are a potentially resource-efficient way to facilitate lifestyle changes (Klimis et al., 2018; Zangger et al., 2023). A simple and accessible mode of delivery is text message interventions (Calling and Thulesius, 2022). Meta-analyses have suggested that text message interventions for patients with hypertension can be effective, but there was substantial heterogeneity between the included studies (David et al., 2022; Siopis et al., 2023; Tam et al., 2021). Interventions that specifically evaluate text messages to patients with hypertension in primary care in high-income countries are sparse. Research to identify mediators and moderators of effect has been called for (Siopis et al., 2023).

Behavior change theories can help us to understand, predict, and change human behavior (Glanz and Bishop, 2010). Such theories have to some extent been used in the design of digital interventions for cardiovascular prevention (Akinosun et al., 2021; Shariful Islam et al., 2019; Winter et al., 2016). However, to our knowledge, prior studies have scarcely applied behavior change theories to understand how digital interventions affect cardiovascular prevention.

**Fig. 1:**
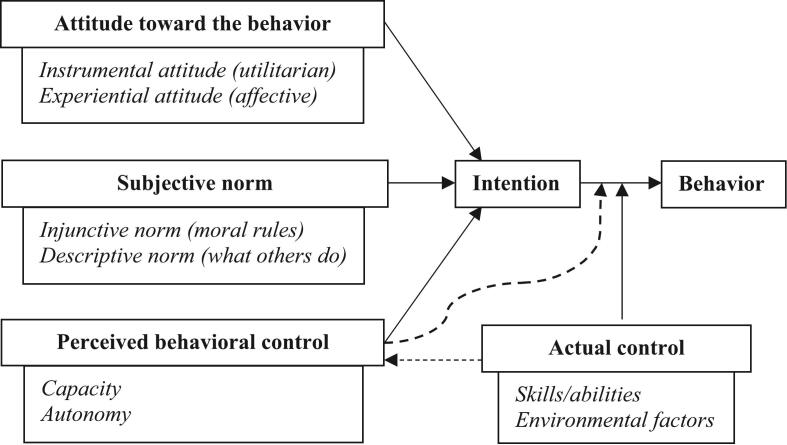
The Theory of Planned Behavior, adapted from Fishbein and Ajzen (Ajzen, 1991, Fishbein and Ajzen, 2010).

A well-established theory of behavior change is the Theory of Planned Behavior (Ajzen, 1991; Fishbein and Ajzen, 2010). According to this theory, three predictors affect an individual's behavioral intention and thus if a behavior is performed: attitude towards the behavior, subjective norms, and perceived behavioral control (Ajzen, 1991). As shown in Fig. 1, each predictor can be further specified into two aspects, and the degree to which intention is translated into behavior is moderated by actual control (Ajzen, 1991; Fishbein and Ajzen, 2010). An appealing clinical application of the theory would be to find one or a few simple questions that could be used to evaluate if a patient would be likely to make a lifestyle change based on an intervention.

From 2020 to 2023, we conducted a randomized controlled trial in which health-promoting text messages were sent to patients with hypertension in Swedish primary care (Primary Care Usage of Health Promoting Messages [PUSHME], ClinicalTrials.gov identifier: NCT04407962). The aim of this study was to investigate whether health-promoting text messages sent to patients with hypertension in primary care could affect lifestyle habits, and if the Theory of Planned Behavior could be used to identify moderators of intervention effects.

## 2 Method

### 2.1 Design

We conducted a multi-center randomized controlled trial to evaluate the effect of health-promoting text messages sent to patients with hypertension in Swedish primary care. A pilot trial had shown the feasibility of the study protocol (Bolmsjo et al., 2020). Participants were allocated 1:1 to receive text messages in addition to treatment as usual, or only treatment as usual. Baseline data and effects on blood pressure (primary outcome measure) have been analyzed separately (Borgstrom Bolmsjo et al., 2025). This study analyzes the behavioral effects of the text messages. The manuscript was prepared according to the Consolidated Standards of Reporting Trials (CONSORT) guidelines (Schulz et al., 2010).

### 2.2 Participants

Participants were randomly selected from the patient register at 10 primary health care centers in southern Sweden. The primary health care centers had mixed locations from urban to rural and were of different sizes. Invitation letters were sent by postal mail, and one to two weeks thereafter a research nurse contacted the potential participants via telephone to offer more information and to schedule a baseline visit if the patient consented. To be eligible, patients had to have a diagnosis of hypertension (International Classification of Diseases 10th Revision code I10.9), be 40–85 years of age, and own a smartphone. Exclusion criteria were blood pressure > 180/110 mmHg or systolic blood pressure < 120 mmHg at baseline, serious illness with life expectancy below one year, or predicted inability to follow the study protocol (e.g. language, cognition).

### 2.3 Intervention

Participants in the intervention group received four health-promoting text messages per week for six months, including lifestyle advice, information, and links to educational material. The text messages were created by the researchers using Swedish national guidelines and expert advice from the regional health care administration. The messages were grouped into four areas: general cardiovascular health (including alcohol), physical activity, diet, and smoking (for smokers only). Participants received at least one message from each group per week.

### 2.4 Outcomes

#### 2.4.1 Lifestyle habits

**Fig. 2:**
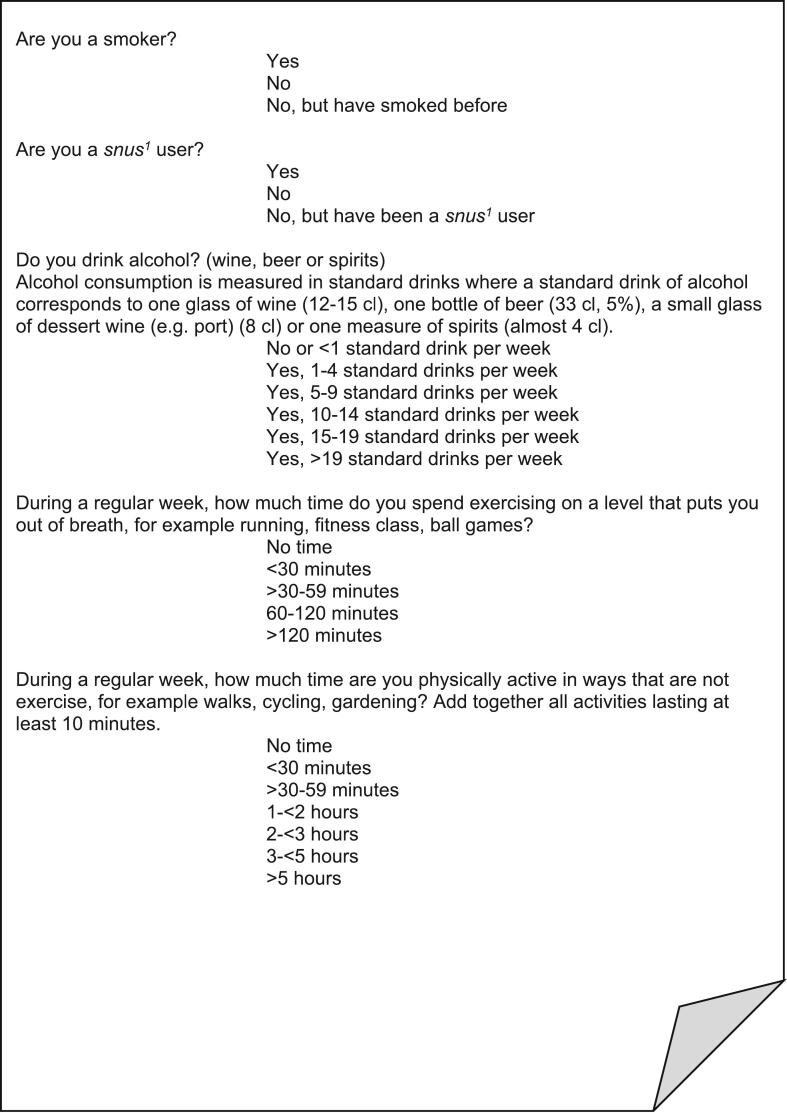
Questionnaire items on lifestyle habits used in a six-month health-promoting text message intervention for patients with hypertension in Swedish primary care conducted in 2020–2023. ^1^ A Swedish moist tobacco powder that is put under the upper lip.

Self-reported measures of lifestyle habits (tobacco use, alcohol use, and physical activity) were collected through a questionnaire at a baseline visit with a study nurse before the trial commenced, and at a follow-up visit after six months of receiving text messages (Fig. 2). To analyze changes in unhealthy habits, we dichotomized the reported habits. The participants who marked “yes” on smoking were defined as smokers. For alcohol use, the cut-off for dichotomization was set at more than four standard drinks per week according to previously established associations between alcohol intake and hypertension (Cecchini et al., 2024; World Health Organization, 2024). We also analyzed the full range of responses on alcohol use. To measure physical activity, we used two validated questions from the Swedish National Board of Health and Welfare, covering moderate-intensity and vigorous-intensity physical activity (Kallings, 2014; Olsson et al., 2016). Weekly physical activity minutes were calculated using the formula (2*vigorous + moderate activity) with midpoint values of the answer options (Kallings, 2014; Olsson et al., 2016). In accordance with national and international recommendations, the cut-off for physical inactivity was set at <150 activity minutes per week (Strain et al., 2024). To analyze the overall physical activity level, we used the full range of weekly physical activity minutes. Finally, we summarized all dichotomized lifestyle habits (smoking, alcohol use above four standard units/week, and physical activity <150 min/week) in a lifestyle index of number of unhealthy habits.

#### 2.4.2 Theory of Planned Behavior

Predictors of behavioral change according to the Theory of Planned Behavior (Fig. 1) were collected at baseline. Using a manual, we designed a pragmatic questionnaire by selecting, translating, and adapting the questionnaire items that were most relevant to our study format and population (Table A1) (Francis et al., 2004). Participants rated their level of agreement or disagreement on a seven-point Likert scale ranging from “completely disagree” to “completely agree”. For predictors covered by several sub-items, internal consistency was tested with Cronbach alpha, and composite scores were calculated if Cronbach alpha was ≥0.7.

### 2.5 Sample size

Sample size was determined for the primary outcome measure, which was change in blood pressure (Borgstrom Bolmsjo et al., 2025). Using data from the pilot study, we estimated a four mmHg difference in blood pressure change between the text message and control groups, a standard deviation of 13 mmHg, and a 10 % dropout rate (Bolmsjo et al., 2020). Assuming a two-sided test, an *α* level of 0.05, and 80 % power rendered a sample size of 186 patients in each arm.

### 2.6 Randomization and blinding

Randomization was performed by a researcher not connected to the patient or the site, after completion of the baseline visit. A computer-generated predefined block sequence was used for each study site, and the patient was informed via postal mail. The study nurses and the patients' primary care physicians were blinded to the allocation.

### 2.7 Statistical methods

The data were analyzed according to the intention-to-treat principle. We compared lifestyle habits between the intervention and control groups at follow-up with adjustment for baseline measures. Lifestyle habits of patients lost to follow-up were imputed with the last observation carried forward, while missing items in the Theory of Planned Behavior questionnaire were imputed with the median. As a sensitivity analysis, a complete case analysis was performed. We also conducted subgroup analyses of age, sex, and educational level interactions on intervention effects. For the subgroup analysis, the Bonferroni correction was used to adjust the significance level for multiple comparisons.

Dichotomous outcome variables (smoker or not, alcohol use with cut-off more than four drinks/week, physical activity with cut-off <150 min per week, and lifestyle index of zero or at least one unhealthy habit) were analyzed using binary logistic regression with intervention status and corresponding baseline measure as covariates. For analysis of the full range of alcohol use (six categories), and for the full lifestyle index (four categories), we employed ordinal logistic regression. For analysis of the full range of weekly physical activity minutes, parametric methods were deemed appropriate (Norman, 2010; Rhemtulla et al., 2012). Consequently, differences in mean physical activity at follow-up were analyzed using analysis of covariance with intervention status as fixed effect and corresponding baseline measure as covariate.

Parametric methods were also used to analyze predictors of behavioral change according to the Theory of Planned Behavior (Norman, 2010; Sullivan and Artino Jr., 2013). For analysis of baseline data, Pearson's correlation coefficient was calculated as a measure of association between each predictor and behavioral intention for lifestyle change. Multiple linear regression, with adjustment for age and sex, was used to quantify the relative effect of the predictors on behavioral intention for lifestyle change. To analyze whether the behavioral predictors could explain lifestyle change in the total cohort, we included perceived behavioral control (capacity and autonomy) and behavioral intention as covariates in the binary logistic regression analyses of intervention effects on lifestyle habits (Fig. 1). To analyze whether intervention effects could be modified by the behavioral predictors, we added an interaction effect of intervention status and each predictor in the regression analyses. The Nagelkerke R^2^ value and the Hosmer-Lemeshow test were used as measures of goodness of fit. The data were analyzed using IBM SPSS Statistics (Version 29).

### 2.8 Ethics

All procedures were performed in compliance with relevant laws and institutional guidelines. The study was approved by the Swedish Ethical Review Authority (date: 22 April 2020, reference number: 2019–06361). The safety and privacy rights of human subjects have been observed. An informed consent form was collected at the baseline visit. An external expert in good clinical practice ensured adherence to the study protocol at all study sites. Analyses were performed on pseudonymized data.

## 3 Results

### 3.1 Recruitment and participants

Participant flow is presented in Fig. A1. Starting in September 2020, a total of 1162 patients with hypertension were approached regarding participation in the study. In total 663 patients declined to participate, and 98 did not meet the inclusion criteria. The remaining 401 patients were randomized to intervention (*n* = 193) and control (*n* = 208). The last patient was included in December 2022 as the number of participants required according to the power calculation had then been reached. The last follow-up visit was conducted in June 2023. Participant baseline characteristics are shown in Table 1. Characteristics of the participants lost to follow-up are reported in Table A2. The dropout rate was 7.2 % (29/401). Most of the participants who dropped out (24/29) had been randomized to the control group.

**Table 1:** Baseline characteristics of study participants in a six-month health-promoting text message intervention for patients with hypertension in Swedish primary care conducted in 2020–2023, by treatment group and total.

	Control (*n* = 208), *n* (%)	Text messages (*n* = 193), *n* (%)	Total (*N* = 401), *n* (%)
Women	103 (49.5)	88 (45.6)	191 (47.6)
Age (years), mean (SD)	69.0 (9.8)	68.2 (8.9)	68.6 (9.4)
Upper secondary or higher education	153 (73.6)	139 (72.0)	292 (72.8)
Heredity of high blood pressure	136 (65.4)	128 (66.3)	264 (65.8)
>5 years with hypertension diagnosis	143 (68.8)	129 (66.8)	272 (67.8)
Previous cardiovascular disease	30 (14.4)	28 (14.5)	58 (14.5)
Diabetes mellitus	29 (13.9)	36 (18.7)	65 (16.2)
Body mass index (kg/m^2^), mean (SD)	28.7 (5.1)	28.4 (4.8)^1^	28.6 (5.0)^1^
Systolic blood pressure (mmHg), mean (SD)	140.5 (13.1)	140.4 (13.0)	140.5 (13.0)
Diastolic blood pressure (mmHg), mean (SD)	84.6 (10.6)	83.5 (9.4)	84.1 (10.0)
HbA1c (mmol/mol), mean (SD)	39.3 (6.6)	40.1 (6.8)	39.6 (6.7)
Non-HDL cholesterol (mmol/L), mean (SD)	3.5 (1.1)^1^	3.4 (1.2)	3.4 (1.1)^1^
Good or very good self-rated health	144 (69.2)	136 (70.5)	280 (69.8)
Behavioral intention for healthy lifestyle^6^	140 (67.3)	134 (69.4)	274 (68.5)
Medications			
Number of medications, median (Q1, Q3)	4 (2, 6)	4 (2, 6)	4 (2, 6)
Number antihypertensives, median (Q1, Q3)	2 (1,2)	2 (1, 2)	2 (1, 2)
≥2 antihypertensives	131 (63.0)	114 (59.1)	245 (61.1)
Lipid-lowering medication	94 (45.2)	89 (46.1)	183 (45.6)
Lifestyle habits			
Current smoker	7 (3.4)	10 (5.2)	17 (4.2)
Current *snus*^2^ user	16 (7.7)	14 (7.3)	30 (7.5)
Alcohol >4 standard drinks/week	57 (27.4)	47 (24.4)	104 (25.9)
Standard drinks/week^3^, median (Q1, Q3)	1–4 (<1, 5–9)	1–4 (<1, 1–4)	1–4 (<1, 5–9)
Physical activity <150 min/week^4^	66 (31.7)	62 (32.1)	128 (31.9)
Physical activity minutes/week^4^, mean (SD)	237.5 (153.3)	243.7 (151.3)	240.5 (152.2)
Lifestyle index			
No unhealthy lifestyle habits^5^	94 (45.2)	93 (48.2)	187 (46.6)
1 unhealthy lifestyle habit^5^	98 (47.1)	85 (44.0)	183 (45.6)
2 unhealthy lifestyle habits^5^	16 (7.7)	11 (5.7)	27 (6.7)
3 unhealthy lifestyle habits^5^	0 (0.0)	4 (2.1)	4 (1.0)

Abbreviations: SD, standard deviation; kg, kilogram; m, meter; mmHg, millimeters of mercury; mmol, millimole; L, liter; Q1, first quartile; Q3, third quartile.

1 One patient's data missing.

2 A Swedish moist tobacco powder that is put under the upper lip.

3 Answer options of standard drinks/week: <1, 1–4, 5–9, 10–14, 15–19, >19.

4 Calculated as (2*vigorous + moderate activity) from mid-point values of answer options with ranges of minutes of activity/week.

5 Smoking, alcohol use >4 standard drinks/week, or physical activity <150 min/week.

6 Likert rating of 5–7 on statement “I plan to make a lifestyle change and/or retain a healthy lifestyle onwards” where 1 = “completely disagree” and 7 = “completely agree”.

### 3.2 Outcomes

#### 3.2.1 Lifestyle habits

As shown in Table 2, there was a significant decrease in the proportion of participants with one or more unhealthy lifestyle habits in the text message group compared to the control group at six months follow-up with adjustment for baseline. Considering the separate lifestyle habits, there were few smokers, and there was no significant change in smoking habits between the text message and control groups. When analyzing the full range of alcohol use, there was a trend towards decreased consumption in the intervention group but no statistically significant difference between the groups (OR 0.60, 95 % CI 0.35–1.01). However, the proportion of participants who consumed more than four standard drinks of alcohol per week decreased significantly in the text message group compared to the control group (OR 0.35, 95 % CI 0.15–0.81). Also, the proportion of physically inactive participants at follow-up was significantly lower in the text message group than in the control group (OR 0.60, 95 % CI 0.37–0.98). Analyzing overall physical activity, there was an increase in mean activity level in the text message group compared to the control group. The increase corresponded to 25.8 (95 % CI 2.7–48.9) minutes of moderate-intensity physical activity (e.g. walking) or 13 min of vigorous-intensity physical activity (e.g. running) per week (Table 2). A complete case analysis did not significantly alter the results. A subgroup analysis of age, sex, and educational level interactions on intervention effects found no such interactions.

**Table 2:** Changes in lifestyle habits during a six-month health-promoting text message intervention for patients with hypertension in Swedish primary care conducted in 2020–2023, comparing intervention and control groups (*N* = 401).

Variable	Control (*n* = 208), *n* (%)	Text messages (*n* = 193), *n* (%)	Text message vs control group at follow-up, adjusted for baseline
			Odds ratio (95 % CI)
Lifestyle index			
≥1 unhealthy habits at baseline	114 (54.8)	100 (51.8)	
≥1 unhealthy habits at follow-up	117 (56.2)	85 (44.0)	0.53 (0.33–0.87)^1^
Number of unhealthy habits at baseline, median (Q1, Q3)	1 (0,1)	1 (0, 1)	
Number of unhealthy habits at follow-up, median (Q1, Q3)	1 (0, 1)	0 (0, 1)	0.50 (0.32–0.79)^2^
Smoking			
Smoker at baseline	7 (3.4)	10 (5.2)	N/A
Smoker at follow-up	5 (2.4)	9 (4.7)	3.60 (0.26–50.33)^3^
Alcohol use			
Alcohol >4 standard drinks/week at baseline	57 (27.4)	47 (24.4)	N/A
Alcohol >4 standard drinks/week at follow-up	58 (27.9)	37 (19.2)	0.35 (0.15–0.81)^4^
Standard drinks/week^5^ at baseline, median (Q1, Q3)	1–4 (<1, 5–9)	1–4 (<1, 1–4)	N/A
Standard drinks/week^5^ at follow-up, median (Q1, Q3)	1–4 (<1, 5–9)	1–4 (<1, 1–4)	0.60 (0.35–1.01)^6^
Physical activity			
Physical activity <150 min/week^7^ at baseline	66 (31.7)	62 (32.1)	N/A
Physical activity <150 min/week^7^ at follow-up	75 (36.1)	54 (28.0)	0.60 (0.37–0.98)^8^
			Mean difference (95 % CI)
Physical activity minutes/week^7^ at baseline, mean (SD)	237.5 (153.3)	243.7 (151.3)	N/A
Physical activity minutes/week^7^ at follow-up, mean (SD)	234.6 (149.2)	264.2 (152.4)	25.8 (2.7–48.9)^9^

Abbreviations: CI, confidence interval; Q1, first quartile; Q3, third quartile; N/A, not applicable; SD, standard deviation.

1 Binary logistic regression with baseline unhealthy habits as covariate.

2 Ordinal logistic regression with baseline unhealthy habits as covariate.

3 Binary logistic regression with baseline smoking status as covariate.

4 Binary logistic regression with baseline alcohol use as covariate.

5 Answer options of standard drinks of alcohol/week: <1, 1–4, 5–9, 10–14, 15–19, >19.

6 Ordinal logistic regression with baseline alcohol use as covariate.

7 Calculated as (2*vigorous + moderate activity) from mid-point values of answer options with ranges of minutes of activity/week.

8 Binary logistic regression with baseline physical activity level as covariate.

9 One-way analysis of covariance with baseline physical activity minutes as covariate.

#### 3.2.2 Theory of Planned Behavior

Participant ratings on the Theory of Planned Behavior questionnaire are detailed in Table A3. The response rate was 100 %. Most participants reported a positive intention towards lifestyle change, with 68.3 % (274/401) agreeing that they intended to make a lifestyle change or retain a healthy lifestyle onwards (Table A3). The perceived capacity to make a lifestyle change showed the strongest correlation with behavioral intention to make a lifestyle change (Table A4). However, the regression model's predictive ability was weak, also when all predictors were added, thus explaining only 36 % of the variance in behavioral intention (Table A5).

Analyzing whether the predictors according to the Theory of Planned Behavior could explain lifestyle change in the total cohort during the intervention, we found a statistically significant increase in the odds of alcohol use above four standard drinks per week for participants with higher ratings on perceived behavioral control in the form of capacity (OR 1.33, 95 % CI 1.003–1.77), but no other correlations (Table A6). There were no interaction effects between the behavioral predictors and intervention status. A complete case analysis did not significantly alter the results.

## 4 Discussion

This randomized controlled trial, which investigated the effects of health-promoting text messages to a Swedish primary care population with hypertension, showed a potentially clinically significant effect on lifestyle habits. The text message group had a statistically significant decrease in the proportion of participants with alcohol use above four standard drinks per week, and in the proportion of participants that were physically inactive compared to the control group. The effect could not be explained or predicted by a pragmatic adaptation of the Theory of Planned Behavior.

A major strength of this study is its multi-center randomized controlled trial design. No similar intervention using text messages has previously been performed in Swedish primary care, and we included 401 individuals with a low dropout rate. Nonetheless, most of the participants who dropped out were in the control group, hence creating potential bias. However, analysis according to the intention-to-treat principle and a complete case analysis produced equivalent results, and improvement of lifestyle habits was largely consistent regarding alcohol use and physical activity, which indicates a relative robustness of results. With regard to generalizability, a healthy volunteer bias must be considered. Contamination bias may also have been present, as the participants could share their text messages. This would likely have favored the control group. Notably, contamination would be a strength when it comes to clinical practice as the intervention may then reach more people. Regarding data sources, the measures of lifestyle habits were self-reported, which is a common practice, but decreases precision and may explain the relatively wide confidence intervals in the analysis (Bulto et al., 2024; Limb et al., 2019). Further, we provided text message advice on diet but did not include a measure of dietary habits as part of the data collection. This was due to the difficulty of finding a brief but satisfactory method to measure dietary habits. Finally, a more extensive and validated Theory of Planned Behavior questionnaire could have increased the possibility of identifying behavioral predictors of intervention effects.

Taking the limitations into account, our analysis of lifestyle habits suggests that health-promoting text messages can have beneficial effects in Swedish primary care patients with hypertension. This is in line with prior studies of text messages for cardiovascular prevention that have generally shown modest but measurable effects (David et al., 2022; Klimis et al., 2018; Klimis et al., 2021; Siopis et al., 2023; Tam et al., 2021). A meta-meta-analysis of digital health interventions for lifestyle habits concluded that interventions were generally effective for behavioral change including for physical activity, where the analysis showed a mean difference of 55.1 (95 % CI 13.8–96.4) minutes/week in moderate-to-vigorous activity (Singh et al., 2024). Our results fall within the lower half of that range. The decrease in the proportion of physically inactive participants in our text message group compared to the control group also indicated a small effect size (Maher et al., 2013). These effects can still be considered clinically significant, as even small changes of replacing sedentary time with physical activity have been shown to reduce cardiovascular mortality (Dohrn et al., 2018).

Regarding alcohol use, a linear association has been observed with the risk for hypertension starting already at one standard drink per day, with roughly 10 % increases in the relative risk of hypertension for each standard drink (Cecchini et al., 2024). The reduction in alcohol use above four standard drinks/week in our text message group, with an odds ratio indicating a medium size effect, is thus of clinical relevance (Maher et al., 2013). However, analyzing the full range of alcohol use, the effect size was small and did not attain the established level of statistical significance (Maher et al., 2013).

Our results thus indicate a potential but modest clinical effect. Nonetheless, a modest effect may still be of value, particularly if the population is large and several lifestyle habits are improved (Fu et al., 2020; Silva et al., 2024; Zhou et al., 2021). With limited health care resources, simple and widely applicable interventions are needed (Albarqouni et al., 2023; Zhou et al., 2021). In this respect, similar to synthesized evidence, our interaction analyses did not show any difference in intervention effects between different subgroups. This suggests that text messages and other digital health interventions can be applied across subpopulations (Singh et al., 2024). However, there might be room for further optimization by increased individualization of the text messages (Bolmsjo et al., 2020; Glock et al., 2023; Redfern et al., 2016).

Regarding our analysis using behavioral change theory, the predictors according to the Theory of Planned Behavior showed a weak predictive ability for behavioral intention for lifestyle change as measured at baseline. There were no clinically significant correlations between the behavioral predictors and self-reported lifestyle change. A possible explanation is that our pragmatic questionnaire did not measure the components of the theory with enough precision. It may also be that the participants did not have sufficient actual control (skills/abilities and control over environmental factors) to follow through with their intended lifestyle changes (Ajzen, 1991, Fishbein and Ajzen, 2010).

In conclusion, health-promoting text messages could be offered to Swedish primary care patients with hypertension as part of the effort to improve lifestyle habits. Future research could investigate if some types of text messages, such as individualized versions, are more effective. A more detailed application of the Theory of Planned Behavior, possibly focusing on perceived capacity to make a lifestyle change, may serve a purpose in this aspect. Future studies may also consider using composite measures of cardiovascular risk as a primary outcome measure, to mirror total effects of the intervention. Finally, studies regarding the long-term effects of text message lifestyle interventions should be considered.

## Funding

This work was supported by the Swedish Heart Lung Foundation (grant numbers 20200228, 20230218), the Swedish Southern Health Care Region and Swedish governmental funding of clinical research (ALF) awarded to Susanna Calling. The funders had no role in study design, data collection, or analysis.

## CRediT authorship contribution statement

**Hanna Glock:** Writing – review & editing, Writing – original draft, Visualization, Methodology, Formal analysis, Data curation, Conceptualization. **Amanda Björk Javanshiri:** Writing – review & editing, Conceptualization. **Beata Borgström Bolmsjö:** Writing – review & editing, Supervision, Resources, Project administration, Methodology, Investigation, Data curation, Conceptualization. **Ulf Jakobsson:** Writing – review & editing, Supervision, Methodology, Conceptualization. **Veronica Milos Nymberg:** Writing – review & editing, Supervision, Resources, Project administration, Methodology, Investigation, Data curation, Conceptualization. **Moa Wolff:** Writing – review & editing, Supervision, Resources, Project administration, Methodology, Investigation, Data curation, Conceptualization. **Susanna Calling:** Writing – review & editing, Supervision, Resources, Project administration, Methodology, Investigation, Funding acquisition, Data curation, Conceptualization.

## Declaration of competing interest

The authors declare that they have no known competing financial interests or personal relationships that could have appeared to influence the work reported in this paper.

## Data Availability

Data will be made available on request.

## Appendix A Supplementary data

CONSORT checklist Image 1

Supplementary tables and figures Image 2
