# Supplementary material for: Promoting lifestyle change through text messages to patients with hypertension: A randomized controlled trial in Swedish primary care
Source: Prev Med Rep. 2025 Feb 17;51:103009. doi: 10.1016/j.pmedr.2025.103009 (PMC11891742; doi:10.1016/j.pmedr.2025.103009)
Supplement: Supplementary file 2 — Supplementary tables and figures [file mmc2.docx]

**Table A1** Questionnaire inspired by the Theory of Planned Behavior

Authors’ note: The behavioral predictor and *the aspect of the predictor* that each question covers are provided below, but were not shown to the participants.

Here are some short questions regarding your experiences and attitudes toward lifestyle change.

Attitude toward the behavior

*Instrumental attitude*

A lifestyle change would have very positive effects on my health.

A lifestyle change should be prioritized in my life right now.

*Experiential attitude*

I feel that I receive sufficient support from the healthcare system to be able to make a lifestyle change.

Subjective norm

*Injunctive norm*

It is expected of me to make a lifestyle change to attain better health.

Perceived behavioral control

*Capacity*

I am convinced that I can make a lifestyle change to improve my health.

*Autonomy*

To be able to make a lifestyle change is completely beyond my control.

Intention

I plan to make a lifestyle change and/or maintain a healthy lifestyle onwards.

For each statement, participants rated their agreement on the following Likert scale:

“1 Completely disagree

2

3

4

5

6

7 Completely agree”

Randomized (*n*=401)

Excluded (*n*=101):

BP >180/110 mmHg (*n*=19)

SBP <120 mmHg (*n*=48)

Other/declined to participate (*n*=34)

Lost to follow-up (*n*=24)^1^

Lost to follow-up (*n*=5)

Allocated to control (*n*=208)

Allocated to intervention (*n*=193)

Enrollment

Allocation

Follow-up

Analysis

Excluded (*n*=660):

Not meeting inclusion criteria (*n*=31):

No smartphone/unable to receive

text messages (*n*=14)

Serious illness (*n*=14)

Language difficulties (*n*=3)

Other/declined to participate (*n*=629)

Scheduled for baseline visit (*n*=502)

Assessed for eligibility (*n*=1162)

**Figure A1** Participant flow chart of a six-month health-promoting text message intervention for patients with hypertension conducted in Swedish primary care in 2020-2023.

Abbreviations: BP, blood pressure; mmHg, millimeters of mercury; SBP; systolic blood pressure.

^1^For one patient, biological measurement data was available at follow-up, but not questionnaire data. That patient is reported as “lost to follow-up”.

^2^Performed as a sensitivity analysis.

Analyzed by intention-to-treat (*n*=193)

Complete case analysis^2^ (*n*=188)

Analyzed by intention-to-treat (*n*=208)

Complete case analysis^2^ (*n*=184)

**Table A2** Baseline characteristics of participants lost to follow-up and total in a six-month health-promoting text message intervention for patients with hypertension in Swedish primary care conducted in 2020-2023.

|  | **Lost to follow-up (*n*=29)^1^** | | | **Total**  **(*N*=401),**  ***n* (%)** | |
| --- | --- | --- | --- | --- | --- |
|  | **Control**  **(*n*=24),**  ***n* (%)** | ***Text messages (n=5)^1^,***  ***n (%)*** | **Total missing**  **(*n*=29)^1^,**  ***n* (%)** | |  |
| Women | 14 (58.3) | 3 (60.0) | 17 (58.6) | | 191 (47.6) |
| Age (years), mean (SD) | 70.4 (9.3) | 69.8 (10.6) | 70.3 (9.3) | | 68.6 (9.4) |
| Upper secondary or higher education | 18 (75.0) | 2 (40.0) | 20 (69.0) | | 292 (72.8) |
| Heredity of high blood pressure | 12 (50.0) | 4 (80.0) | 16 (55.2) | | 264 (65.8) |
| >5 years with hypertension diagnosis | 16 (66.7) | 3 (60.0) | 19 (65.5) | | 271 (67.6) |
| Previous cardiovascular disease | 4 (16.7) | 2 (40.0) | 6 (20.7) | | 58 (14.5) |
| Diabetes mellitus | 5 (20.8) | 0 (0) | 5 (17.2) | | 65 (16.2) |
| Body mass index (kg/m^2^), mean (SD) | 28.4 (6.6) | 28.9 (2.2) | 28.5 (6.0) | | 28.6 (5.0)^5^ |
| Systolic blood pressure (mmHg), mean (SD) | 144.1 (14.1) | 133.5 (15.0) | 142.3 (14.5) | | 140.5 (13.0) |
| Diastolic blood pressure (mmHg), mean (SD) | 85.6 (11.9) | 88.4 (8.0) | 86.1 (11.2) | | 84.1 (10.0) |
| HbA1c (mmol/mol), mean (SD) | 39.3 (8.2) | 36.4 (4.6) | 38.8 (7.7) | | 39.6 (6.7) |
| Non-HDL cholesterol (mmol/L), mean (SD) | 3.4 (1.0) | 3.2 (1.1) | 3.3 (1.0) | | 3.4 (1.1)^5^ |
| Current smoker | 0 (0) | 2 (40.0) | 2 (6.9) | | 17 (4.2) |
| Current *snus*^2^ user | 3 (12.5) | 0 (0) | 3 (10.3) | | 30 (7.5) |
| Alcohol >4 standard drinks/week | 9 (37.5) | 1 (20.0) | 10 (34.5) | | 104 (25.9) |
| Physical activity <150 minutes/week^3^ | 7 (29.2) | 2 (40.0) | 9 (31.0) | | 128 (31.9) |
| Physical activity minutes/week^3^, mean (SD) | 233.1 (147.9) | 195.0 (167.0) | 227.6 (148.9) | | 240.5 (152.2) |
| Good or very good self-rated health | 15 (62.5) | 2 (40.0) | 17 (58.6) | | 280 (69.8) |
| Behavioral intention for healthy lifestyle^4^ | 16 (66.7) | 2 (40.0) | 18 (62.1) | | 275 (68.6) |

Abbreviations: SD, standard deviation; kg, kilogram; m, meter, mmHg, millimeters of mercury; mmol, millimole; L, liter.

^1^For one patient, biological measurement data was available at follow-up, but not questionnaire data. That patient is reported as “missing”.

^2^A moist tobacco powder that is put under the upper lip.

^3^Calculated as (2*vigorous + moderate activity) from mid-point values of answer options with ranges of minutes of activity/week.

^4^Likert rating of 5-7 on statement “I plan to make a lifestyle change and/or retain a healthy lifestyle onwards” where 1 = “completely disagree” and 7 = “completely agree”.

**Table A3** Items and constructs of a pragmatic Theory of Planned Behavior questionnaire at baseline of a six-month health-promoting text message intervention for patients with hypertension in Swedish primary care conducted in 2020-2023 *(N*=401).

| **Items and constructs** | **Likert ratings^1^, *n* (%)** | | | | | | | | | | | **Median (Q1, Q3)** | | **Mean (SD)** | | **Cron-bach alpha** |
| --- | --- | --- | --- | --- | --- | --- | --- | --- | --- | --- | --- | --- | --- | --- | --- | --- |
|  | **1** | **2** | **3** | **4** | **5** | **6** | **7** | **1-4** | **5-7** | **Missing** |  | |  | |  | |
| **Items** |  |  |  |  |  |  |  |  |  |  |  | |  | |  | |
| 1. Instrumental attitude | 25 (6.2) | 24 (6.0) | 33 (8.2) | 76 (19.0) | 82 (20.4) | 56 (14.0) | 103 (25.7) | 158 (39.4) | 241 (60.1) | 2 (0.5) | 5.0 (4.0, 7.0) | | 4.9 (1.8) | |  | |
| 2. Instrumental attitude | 37 (9.2) | 34 (8.5) | 49 (12.2) | 76 (19.0) | 67 (16.7) | 49 (12.2) | 89 (22.2) | 196 (48.9) | 205 (51.1) | 0 (0) | 5.0 (3.0, 6.0) | | 4.5 (1.9) | |  | |
| 3. Experiential attitude | 42 (10.5) | 28 (7.0) | 43 (10.7) | 88 (21.9) | 47 (11.7) | 50 (12.5) | 98 (24.4) | 201 (50.1) | 195 (48.6) | 5 (1.2) | 4.0 (3.0, 6.0) | | 4.6 (2.0) | |  | |
| 4. Subjective norm | 66 (16.5) | 36 (9.0) | 32 (8.0) | 65 (16.2) | 69 (17.2) | 41 (10.2) | 89 (22.2) | 199 (49.6) | 199 (49.6) | 3 (0.7) | 4.5 (2.0, 6.0) | | 4.3 (2.1) | |  | |
| 5. Control: capacity | 17 (4.2) | 25 (6.2) | 41 (10.2) | 53 (13.2) | 77 (19.2) | 62 (15.5) | 124 (30.9 | 136 (33.9) | 263 (65.9) | 2 (0.5) | 5.0 (4.0, 7.0) | | 5.1 (1.8) | |  | |
| 6. Control: autonomy^2^ | 16 (4.0) | 10 (2.5) | 27 (6.7) | 29 (7.2) | 33 (8.2) | 58 (14.5) | 224 (55.9) | 82 (20.4) | 315 (78.6) | 4 (1.0) | 7.0 (5.0, 7.0) | | 5.8 (1.7) | |  | |
| 7. Behavioral intention | 24 (6.0) | 25 (6.2) | 25 (6.2) | 52 (13.0) | 73 (18.2) | 59 (14.7) | 142 (35.4) | 126 (31.4) | 274 (68.3) | 1 (0.2) | 6.0 (4.0, 7.0) | | 5.2 (1.9) | |  | |
| **Constructs** |  |  |  |  |  |  |  |  |  |  |  | |  | |  | |
| Instrumental attitude (1-2) |  |  |  |  |  |  |  |  |  | 2 (0.5) | 5.0 (3.5, 6.0) | | 4.7 (1.8) | | 0.89 | |
| Attitude toward the behavior (1-3) |  |  |  |  |  |  |  |  |  |  |  | |  | | 0.64 | |
| Perceived behavioral control (5-6) |  |  |  |  |  |  |  |  |  |  |  | |  | | 0.29 | |

Abbreviations: Q1, first quartile; Q3, third quartile, SD, standard deviation.

^1^Ratings range from 1 = “completely disagree” to 7 = “completely agree”.

^2^Item with reverse scoring, reversed for presentation and analysis.

**Table A4** Correlations between predictors of lifestyle change at baseline in a six-month health-promoting text message intervention for patients with hypertension in Swedish primary care conducted in 2020-2023 (*N*=401).

| **Predictor** | **Experiential attitude** | | **Subjective norm** | | **Control: capacity** | | **Control: autonomy** | | **Behavioral intention** | |
| --- | --- | --- | --- | --- | --- | --- | --- | --- | --- | --- |
|  | R^1^ | *P* | R^1^ | *P* | R^1^ | *P* | R^1^ | *P* | R^1^ | *P* |
| Instrumental attitude | 0.18 | <.001 | 0.61 | <.001 | 0.60 | <.001 | -0.02 | .73 | 0.49 | <.001 |
| Experiential attitude |  |  | 0.27 | <.001 | 0.29 | <.001 | -0.01 | .81 | 0.16 | .002 |
| Subjective norm |  |  |  |  | 0.47 | <.001 | -0.03 | .55 | 0.41 | <.001 |
| Control: capacity |  |  |  |  |  |  | 0.17 | <.001 | 0.55 | <.001 |
| Control: autonomy |  |  |  |  |  |  |  |  | 0.11 | .03 |

^1^Pearson’s correlation coefficient.

**Table A5**  Multiple linear regression analysis of behavioral intention for lifestyle change according to the Theory of Planned Behavior at baseline in a six-month health-promoting text message intervention for patients with hypertension in Swedish primary care conducted in 2020-2023, adjusted for sex and age (*N*=401).

|  | **Model 1^1^** | **Model 2^2^** | **Model 3^3^** |
| --- | --- | --- | --- |
| **Model statistics** |  |  |  |
| r^2^ | 0.32 | 0.35 | 0.36 |
| Variance inflation factor | 1.1 | 1.7 | 2.2 |
| **Predictors, *B* (95% CI)^4^** |  |  |  |
| Control: capacity | 0.55 (0.46-0.63) | 0.42 (0.32-0.52) | 0.39 (0.31-0.53) |
| Instrumental attitude |  | 0.24 (0.13-0.34) | 0.18 (0.06-0.30) |
| Subjective norm |  |  | 0.10 (0.01-0.19) |
| Experiential attitude |  |  | -0.01 (-0.09 to 0.07) |
| Control: autonomy |  |  | 0.04 (-0.05 to 0.13) |

Abbreviations: *B*, beta coefficient; CI, confidence interval.

^1^Predictor – control: capacity. Outcome – behavioral intention. Adjustment – sex, age.

^2^Predictors – control: capacity, instrumental attitude. Outcome – behavioral intention. Adjustment – sex, age.

^3^Predictors – control: capacity, control: autonomy, instrumental attitude, experiential attitude, subjective norm. Outcome – behavioral intention. Adjustment – sex, age.

^4^All predictors, and behavioral intention, were measured on a 7-point Likert scale where 1 = “completely disagree” and 7 = “completely agree”.

**Table A6** Effects of intervention status and predictors according to the Theory of Planned Behavior on lifestyle outcome variables at six months follow-up, adjusted for baseline values, in a health-promoting text message intervention for patients with hypertension in Swedish primary care conducted in 2020-2023 (*N*=401).

| Outcome | Predictor variables | | | |
| --- | --- | --- | --- | --- |
|  | Text messages | Behavioral intention^1^ | Control: capacity^1^ | Control: autonomy^1^ |
| Smoker, OR (95% CI)^2^ | 2.51 (0.06-101.54) | 0.97 (0.07-13.02) | 0.28 (0.03-2.55) | 3.15 (0.17-60.17) |
| Alcohol >4 standard drinks/week, OR (95% CI)^3^ | 0.32 (0.14-0.76) | 0.89 (0.69-1.16) | 1.33 (1.003-1.77) | 1.10 (0.83-1.45) |
| Physical activity <150 minutes/ week^4^, OR (95% CI)^5^ | 0.61 (0.37-1.01) | 1.05 (0.89-1.23) | 0.89 (0.76-1.06) | 0.17 (0.79-1.04) |

Abbreviations: OR, odds ratio; CI, confidence interval.

^1^Measured on a 7-point Likert scale where 1 = “completely disagree” and 7 = “completely agree”.

^2^Binary logistic regression with baseline smoking status as covariate.

^3^Binary logistic regression with baseline alcohol use as covariate.

^4^Calculated as (2*vigorous + moderate activity) from mid-point values of answer options with ranges of minutes of activity/week.

^5^Binary logistic regression with baseline physical activity level as covariate.
